# Restricted differentiation potential of progenitor cell populations obtained from the equine superficial digital flexor tendon (SDFT)

**DOI:** 10.1002/jor.22891

**Published:** 2015-04-29

**Authors:** Kate Ann Williamson, Katie Joanna Lee, William James Edward Humphreys, Eithne Josephine Veronica Comerford, Peter David Clegg, Elizabeth Gail Canty‐Laird

**Affiliations:** ^1^ Department of Musculoskeletal BiologyInstitute of Ageing and Chronic DiseaseLeahurst CampusUniversity of LiverpoolChester High RoadNestonCH64 7TEUnited Kingdom; ^2^ School of Veterinary ScienceLeahurst CampusUniversity of LiverpoolChester High RoadNestonCH64 7TEUnited Kingdom; ^3^ The MRC‐Arthritis Research UK Centre for Integrated research into Musculoskeletal Ageing (CIMA)LiverpoolL69 3GAUnited Kingdom

**Keywords:** tendon, progenitor, stem, equine, differentiation

## Abstract

The aim of this study was to characterize stem and progenitor cell populations from the equine superficial digital flexor tendon, an energy‐storing tendon with similarities to the human Achilles tendon, which is frequently injured. Using published methods for the isolation of tendon‐derived stem/progenitor cells by low‐density plating we found that isolated cells possessed clonogenicity but were unable to fully differentiate towards mesenchymal lineages using trilineage differentiation assays. In particular, adipogenic differentiation appeared to be restricted, as assessed by Oil Red O staining of stem/progenitor cells cultured in adipogenic medium. We then assessed whether differential adhesion to fibronectin substrates could be used to isolate a population of cells with broader differentiation potential. However we found little difference in the stem and tenogenic gene expression profile of these cells as compared to tenocytes, although the expression of thrombospondin‐4 was significantly reduced in hypoxic conditions. Tendon‐derived stem/progenitor cells isolated by differential adhesion to fibronectin had a similar differentiation potential to cells isolated by low density plating, and when grown in either normoxic or hypoxic conditions. In summary, we have found a restricted differentiation potential of cells isolated from the equine superficial digital flexor tendon despite evidence for stem/progenitor‐like characteristics. © 2015 The Authors. *Journal of Orthopaedic Research* Published by Wiley Periodicals, Inc. on behalf of Orthopaedic Research Society. J Orthop Res 33:849–858, 2015.

## INTRODUCTION

Sports participation, occupation, and aging increase the risk of tendon injury and degeneration in both humans and animals.^1^, ^2^, ^3^ In the horse, the superficial digital flexor tendon (SDFT) is commonly injured, resulting in lameness and reduced performance, particularly in athletic and racing horses.^3^ SDF tendinopathies are more prevalent with age, and age‐related alterations to both fascicles and the interfascicular matrix of the endotenon alter the response of the SDFT to mechanical loading.^4^, ^5^, ^6^ Tendon injury and age‐related degeneration results in particular patterns of matrix fragmentation that may affect the structural integrity of the tendon extracellular matrix and the microenvironment of tendon cells.^7^

The identification of a population of cells within tendon with stem cell‐like characteristics^8^ holds potential for tendon regeneration. Tendon‐derived stem/progenitor cells (TSPCs) have been identified in human, mouse,^8^ rat,^9^ and rabbit tendon.^10^ TSPCs possess similar properties to mesenchymal stem cells (MSCs) and have been identified by the expression of cell surface and stem‐cell markers, and a capacity for self‐renewal and multi‐lineage differentiation. TSPCs are thought to be tenocyte precursors and can be induced to differentiate into osteocytes, chondrocytes, and adipocytes in vitro and in vivo.^8^, ^9^, ^10^, ^11^, ^12^

In mice, treadmill training has been reported to increase TSPC proliferation and to increase expression of the tenogenic marker scleraxis in epitenon fibroblasts.^13^, ^14^ Cells obtained from the peritenon of mouse Achilles tendon demonstrated decreased clonogenicity compared to the tendon core and limited osteogenic differentiation.^12^ In the equine SDFT, peritenon cells displayed decreased clonogenicity and both osteogenic and adipogenic differentiation, but were reported to have increased proliferation and increased expression of progenitor cell markers.^15^ Tendons are relatively poorly vascularized^16^ and tendon cells reside within a hypoxic environment. Culture of TPSCs in 2% oxygen has been reported to increase proliferation but to reduce multipotency,^17^, ^18^ whereas 5% oxygen reportedly both increases proliferation and maintains stemness.^19^

In this study, we aimed to isolate and characterize TSPCs from non‐diseased samples of the frequently injured equine superficial digital flexor tendon. The equine SDFT primarily acts as an energy store during locomotion and performs a similar role to the human Achilles. We hypothesized that multipotent TSPCs would be present in the equine SDFT as they have previously been identified in tendon from other species. However, injured tendons have limited capacity for healing and tissue regeneration, despite the reported presence of TSPCs in tendon.

## METHODS

### Isolation of TSPCs and Tenocytes

Superficial digital flexor tendon (SDFT) was harvested from equine cadavers (age range 1–22) obtained from a UK abattoir. Tissue samples were grossly normal upon examination. The mid‐substance tendon tissue, without the paratenon/tendon sheath, was dissected in to small pieces and digested overnight at 37°C in 1 mg/ml collagenase II. The resulting cell suspension was strained and then centrifuged at 2,300 rpm for 10 min and the supernatant discarded. The cells were resuspended in complete Dulbecco's modified Eagle's medium (DMEM) (DMEM with GlutaMAX supplemented with 10% foetal calf serum, penicillin [100 U/ml]), streptomycin (100 µg/ml), and amphotericin B [2 µg/ml]), and counted using a haemocytometer. The same batch of foetal calf serum was used for all experiments. For tenocyte isolation, the cells were seeded at 2.8 × 10^4^ cells/cm^2^ and for TSPC isolation the cells were seeded at 10, 80, or 100 cells/cm^2^. The cells were cultured at 37°C, 5% CO_2_ and either 21%, or 5% O_2_ for 10–12 days. Colonies were detached using trypsin and transferred to T25 culture flasks. For differential fibronectin, adhesion cells were seeded at 1,200 cells/cm^2^ after digestion, onto plates previously coated with either 1 or 20 μg/ml human fibronectin, and the media replaced after 20 min. Cells grown on substrates precoated with 20 μg/ml human fibronectin were supplemented with 5 ng/ml FGF‐2. After 6–8 days the cells were confluent and transferred to a T25 culture flask.

### Colony Forming and Tri‐Lineage Differentiation Assays

Tenocytes or TSPCs isolated by low‐density plating were seeded at 10 cells/cm^2^ after the first passage and colonies stained with crystal violet before imaging whole wells with a camera or using a Nikon Eclipse TS100 microcope attached to a Nikon Digital Sight CCD camera. For differentiation assays cell monolayers at passage 2 were cultured for 21 days in osteogenic induction media (complete DMEM containing 100 nM dexamethasone, 10 mM β‐glycerophosphate, and 0.5 μM ascorbic acid) or adipogenic induction media (complete DMEM containing 1 μM dexamethasone, 100 μM indomethacin, 10 μg/ml insulin, and 500 μM isobutylmethylxanthine). Cell pellets were cultured for 21 days in chondrogenic induction media (complete DMEM containing 100 nM dexamethasone, 25 μg/ml ascorbic acid, 10 ng/ml TGF‐β, and ITS+3 media supplement [Sigma‐Aldrich, Gillingham, UK]). Control cells were cultured in complete DMEM with or without phenol red. Cells were stained with alizarin red to assess osteogenic differentiation,^20^ Oil Red O to assess adipogenic differentiation,^21^ and alcian blue for chondrogenic differentiation^22^ as described in the PromoCell MSC application notes. RNA was extracted from all assays to analyze lineage‐specific gene expression.

### RNA Extraction and Quantitative RT‐PCR

RNA was extracted with Trizol and using a cell scraper for cell detachment and by repeated pipetting to disrupt cell pellets. TSPCs were analyzed at passage 1–2. cDNA was synthesized in a 25 μl reaction from 1–2 μg of total RNA by incubation for 5 min at 70°C, 60 min at 37°C, and 5 min at 93°C using M‐MLV reverse transcriptase and random‐hexamer oligonucleotides (Promega Ltd., Southampton, UK). qRT‐PCR was conducted using a GoTaq(R) qPCR Master Mix (Promega). A total of 10 ng of cDNA was amplified in a 25 μl reaction using an AB 7300 Real Time PCR System (Life Technologies Ltd., Paisley, UK). Equine specific gene‐specific primers were used (Table 1) and GAPDH or B2M (Primer Design, Southampton, UK, proprietary sequence) used as internal controls. After an initial denaturation for 10 min at 95°C, 40 PCR cycles were performed consisting of 15 s at 95°C and 1 min at 60°C. Relative gene expression was calculated according to the comparative C_t_ method.^23^

**Table 1:** Primer Sequences

Gene	Forward	Reverse
CD90	TGCTCCGAGACAAACTGGT	CCGAGGTGTGTGAGGGATTG
CD73	CCAGGAAGTGGGGAGAACAC	CCAAGGTAATGGTGCCGTTG
TNC	GTTTCAGATGCCACCCCAGA	AGCCCATAGCTGTTGTTGCT
SCX	TCTGCCTCAGCAACCAGAGA	TCCGAATCGCCGTCTTTC
MKX	GATGACGCTAGTGCAGGTGT	CCCCCTTCGTTCATGTGGTT
EGR‐1	CCACCATGGACAACTACCCT	ATGTCAGGAAAAGACTCTGAGG
DCN	GTCACAGAGCAGCACCTACC	TCACAACCAAGGAACCTTTTAATCC
OCT4	GAGAAGGACGTGGTACGAGTG	GTGCCAGGGGAAAGGATACC
NANOG	CAGGGGATCTTCACCAGTGC	GGAAGGCAGAGGAGAGACAGT
TNMD	ACGTGACCATGTATTGGATCAATC	CACCATCCTCCTCAAAGTCTTGT
THBS4	AATCCTGACAGACCCCACCC	GGTAGCGGAGGATGGCTTTGTT
FABP4	CAGAGGGTCAGAGCACCTTC	GCCCACTCCCACTTCTTTCA
PPARγ	ATGGGTGAAACTCTGGAAGATT	GGTAATTTCTTGTGAAGTGCTTGC
LEP	CATTGAAGCTGTGCCCATCC	AGACTGACTGCGTGTGTGAAA
RUNX2	GTGGACGAGGCAAGAGTTTC	TGAGGCGGTCAGAGAACAAA
OMD	CAAATTCATCAACCCCTGAAA	CTTCATCTGGCTCTTGGTCA
ALP	GGGTAGCAGCCAGTTCAGTT	GGGATCTTTCTCTTTCTCTGGCA
COL1A1	CATGTTCAGCTTTGTGGACCT	TGACTGCTGGGATGTCTTCTT
DCN	GTCACAGAGCAGCACCTACC	TCACAACCAAGGAACCTTTTAATCC
SOX9	AGCAGACACACATCTCCCCC	GCGAGGAATGAGCCTACAAGGT
COL2A1	TGAGCCATGATACGCCTCG	CTCCTTTCTGTCCCTTCGGT
ACAN	GCGGTACGAGATCAACTCCC	CTTGTAGCTGGCGGGGTC
GAPDH	GCATCGTGGAGGGACTCA	GCCACATCTTCCCAGAGG

### Flow Cytometry

TSPCs at passage 1–2 were detached using Accutase (Life Technologies Ltd., Paisley, UK) and counted. Aliquots containing 1 × 10^6^ cells were blocked with 10% normal serum in FACS buffer (2.5% FBS in PBS) for 20 min before washing. Cells were resuspended in either fluorescently conjugated or unconjugated primary antibodies for 45 min at 4°C. Anti‐CD90 (ab225, Abcam, Cambridge, UK), anti‐CD105 (MCA1557A488T, Serotec, Oxford, UK), and anti‐CD73 (550256, BD Biosciences, Oxford, UK) were used in this study. Cells were washed and either analyzed directly (CD105) by flow cytometry (BD Acurri C6 flow cytometer, BD Biosciences, Oxford, UK) or incubated with a secondary antibody for a further 45 min at 4°C (CD90 and CD73) before washing and analysis. Cells in the absence of antibody and in the presence of the secondary antibody only were used as controls. A threshold gating out at least 95.5% of the control cells was used and for samples including primary antibody the percentage of positive cells calculated as that exceeding the threshold.

### Cell Proliferation Assay

Cells were seeded at 133 cells/cm^2^ at passage 2. At 80% confluency the cells were counted and the doubling time calculated.

### Statistical Analysis

Statistical analysis was performed using SigmaPlot 12.5. For pairwise comparisons a *t*‐test was utilized and a Mann–Whitney rank‐sum test used for samples that did not meet the assumption of normality (Shapiro–Wilk test). For comparisons of three samples, a one‐way ANOVA was used. For comparisons across two conditions, a two‐way ANOVA was used. For samples not meeting the assumption of normality for two‐way ANOVA (Shapiro–Wilk test), data were transformed using either a log base 10 or square root transformation so that the resulting data sets were normally distributed. Suitable transformations could not be found for BGLAP in Figure 2M or THBS4 in Figure 5. A Holm–Sidak post‐hoc test was used for two‐way ANOVA results where the resulting p value was less than 0.05.

## RESULTS

### TSPCs from Equine SDF Tendon form Colonies when Plated at Low Density but have Restricted Trilineage differentiation

**Figure 1:**
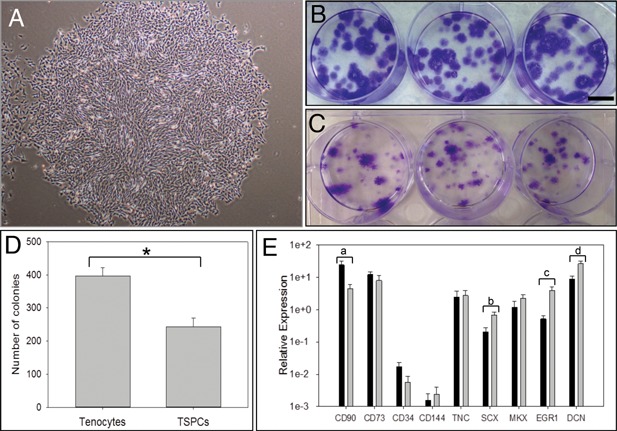
Comparison of the colony forming ability and marker gene expression of tenocytes and tendon‐derived stem/progenitor cells (TSPCs) isolated by low‐density plating. A: Colony formed from TSPCs after low‐density plating. B: Crystal violet staining of colonies formed from TSPCs seeded at 10 cells/cm^2^ after 1 passage. Bar; 1 cm. C: Crystal violet staining of colonies formed from tenocytes seeded at 10 cells/cm^2^ after the first passage. D: Number of colonies formed from tenocytes and TSPCs seeded at 100 cells/cm^2^ after the first passage in T25 flasks. E: Relative expression of stem/progenitor and tenogenic genes as compared to B2M housekeeper in tenocytes (dark gray bars) and TSPCs (light gray bars) isolated by low‐density plating (*n* = 6, mean age 11.0 years, range 1–22 years). Error bars represent SEM. ^*^ *p* = 0.00298, ^a^ *p* = 0.0249, ^b^ *p* = 0.0115, ^c^ *p* = 0.00895, ^d^ *p* = 0.017.

TSPCs isolated from SDF tendon by low‐density plating were able to form colonies after replating, as were tenocytes initially plated at high density (Fig. 1A–C). The number of colonies obtained from tenocytes was higher than that obtained from TSPCs (Fig. 1D). TSPCs had increased expression of the tenogenic markers scleraxis (SCX), early growth response 1 (EGR1), and decorin (DCN) but decreased expression of CD90 (Fig. 1D). Expression of CD34 and CD144 was low in both tenocytes and TSPCs indicating the cell populations are not contaminated with hematopoietic stem cells.

**Figure 2:**
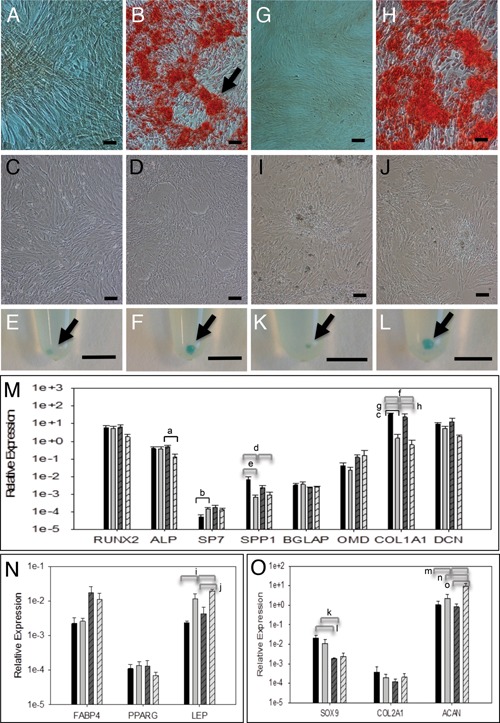
Trilineage differentiation assay for TSPCs isolated by low density plating at 80 cells/cm^2^ (A–F) or by plating onto substrates precoated with 1 μg/ml fibronectin (G–L). A, B and G, H: Representative images of TSPCs grown in control (A, G) and osteogenic induction media (B, H) and stained with Alizarin Red. Sample age 6 years. Bar; 100 μm. C, D and I, J: Representative images of TSPCs grown in control (C, I) and adipogenic induction media (D, J) and stained with Oil Red O. No staining was detected. Sample age 6 years. Bar; 100 μm. E, F and K, L: Representative images of TSPCs pellet cultures grown in control (E, K) and chondrogenic induction media (F, L) and stained with Alcian Blue. Sample ages 8 years (E, F), 6 years (K, L). Bar; 5 mm. M: Relative expression of osteogenic markers as compared to B2M housekeeper for TSPCs isolated by low density plating (*n* = 4, mean age 7.5 years, range 2–14 years) (solid fill) or by differential adhesion to fibronectin (*n* = 3, mean age 7.7 years, range 2–15 years) (hatched fill) and grown in control (dark gray bars) or osteogenic induction media (light gray bars). N: Relative expression of adipogenic markers as compared to B2M housekeeper for TSPCs isolated by low density plating (*n *= 3, mean age 9.7 years, range 6–15 years) (solid fill) or by differential adhesion to fibronectin (*n* = 3, mean age 7.7 years, range 2–15 years) (hatched fill) and grown in control (dark grey bars) or adipogenic induction media (light gray bars). O: Relative expression of chondrogenic markers as compared to B2M housekeeper for TSPCs isolated by low density plating (*n* = 3, mean age 5.3 years, range 2–8 years) (solid fill) or by differential adhesion to fibronectin (*n* = 3, mean age 7.7 years, range 2–15 years) (hatched fill) and grown in control (dark gray bars) or chondrogenic induction media (light gray bars). Error bars represent SEM. ^a–c^Pair‐wise comparisons: ^a^ *p* = 0.0414, ^b^ *p* = 0.0460, ^c^ *p* = 0.0019. ^d–o^2‐way ANOVA: ^d^ *p* = 0.03, ^e^ *p* = 0.037, ^f^ *p* < 0.001, ^g^ *p* < 0.001, ^h^ *p* = 0.003, ^i^ *p* = 0.003, ^j^ *p* = 0.006, ^k^ *p* = 0.014, ^l^ *p* = 0.016, ^m^ *p* = 0.02, ^n^ *p* = 0.018, ^o^ *p* = 0.007.

Growth of equine TSPCs in osteogenic media resulted in Alizarin Red positive nodules (Fig. 2A and B) but no Oil Red O positive lipid droplets could be observed in TSPCs grown in adipogenic media (Fig. 2C and D). Growth of pellet cultures in chondrogenic media resulted in clearly increased pellet size in 2 of 3 cultures (Fig. 2E and F). For TSPCs grown in osteogenic media, there were no significant differences in the expression of osteogenic markers runx2 (RUNX2), alkaline phosphatase (ALP), osteocalcin (BGLAP), or osteomodulin (OMD) in osteogenic as compared to control media (Fig. 2M). Expression of osterix (SP7) and osteopontin (SPP1) was low but either significantly increased (SP7) or decreased (SPP1) between control and osteogenic media. TSPC growth in adipogenic media did not result in increased expression of leptin (LEP), FABP4, or PPARγ (Fig. 2N) and no statistically significant differences in chondrogenic gene expression were found for TSPCs grown in chondrogenic media (Fig. 2O).

We then considered that low density plating may not be a suitable method to isolate equine TSPCs and used differential adhesion to fibronectin as a stem cell isolation method, as previously described for epidermal stem cells and cartilage progenitor cells.^24^, ^25^ Plating onto substrates precoated with 1 μg/ml fibronectin promoted osteogenic differentiation of TSPCs grown in osteogenic media as assessed by Alizarin Red staining (Fig. 2G and H). Adipogenic differentiation was not detected by Oil Red O staining for TSPCs isolated by differential fibronectin adhesion and grown in adipogenic media (Fig. 2I and J) but chondrogenic differentiation was noted in all pellet cultures of the same cells grown in chondrogenic media (Fig. 2K and L). There was no increase in expression of osteogenic markers (Fig. 2M) but there was a significant increase in leptin gene expression (Fig. 2N) for adipogenic cultures and of aggrecan alone for chondrogenic cultures (Fig. 2O).

### Putative TSPCs Can Be Isolated by Differential Adhesion to Fibronectin

**Figure 3:**
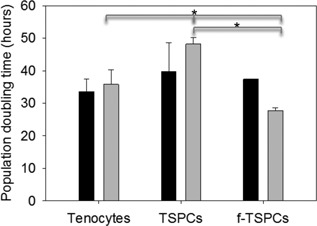
Comparison of population doubling times of TSPCs isolated by low‐density plating and fibronectin adhesion and grown in hypoxic conditions. TSPCs were isolated by plating at 10 cells/cm^2^ or by differential adhesion onto substrates precoated with 20 μg/ml fibronectin (f‐TSPCs), grown in 5% oxygen (light gray bars) and compared to tenocytes. ^*^ *p* = 0.015. Cells grown in 21% (dark gray bars) are shown for comparison however for TSPCs or f‐TSPCs grown in 21% oxygen only two biological replicates were available (age range 1–22 years). Error bars represent SEM.

In subsequent experiments, substrates precoated with 20 μg/ml fibronectin were used and the effect of normoxia (21% oxygen) and hypoxia (5% oxygen) on the characteristics of these putative stem cells (denoted f‐TDCSs) analyzed. In hypoxic conditions, TSPCs isolated by low‐density plating proliferated more slowly than tenocytes or TSPCs isolated by plating onto substrates precoated with 20 μg/ml of fibronectin (Fig. 3).

**Figure 4:**
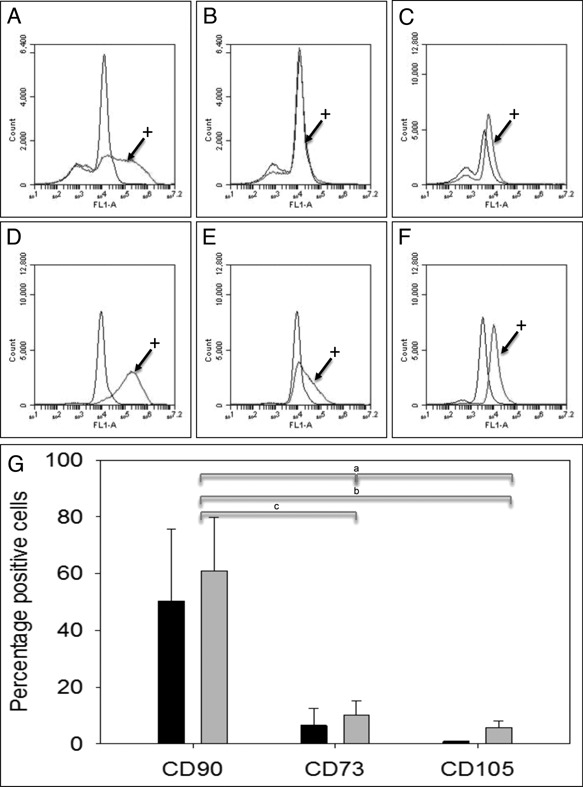
Flow cytometry analysis of cell surface markers CD90, CD73, and CD105 on tendon‐derived stem/progenitor cells isolated by differential adhesion onto substrates precoated with 20 μg/ml fibronectin (f‐TSPCs) and grown in normoxia and 5% hypoxia. A–C: f‐TSPCs (age 1 year) grown in 21% oxygen and incubated with control or antibodies (+) to CD90 (A), CD73 (B), or CD105 (C) for flow cytometry. D–F: f‐TSPCs grown in 5% oxygen and incubated with control or antibodies (+) to CD90 (D), CD73 (E), or CD105 (F) for flow cytometry. ^a^ *p* = 0.024, ^b^ *p* = 0.04, ^c^ *p* = 0.038.

Flow cytometry analysis indicated that f‐TSPCs grown in normoxia or hypoxia expressed the cell surface marker CD90 (Fig. 4A, D, and G). There was some evidence for low expression of CD73 and CD105 in f‐TSPCs grown in hypoxia (Fig. 4B and C, E–G).

**Figure 5:**
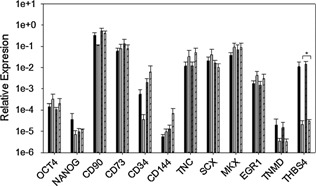
Marker gene expression of tenocytes and tendon‐derived stem/progenitor cells isolated by differential adhesion onto substrates precoated with 20 μg/ml fibronectin (f‐TSPCs). Relative expression of stem/progenitor and tenogenic genes as compared to GAPDH in tenocytes (dark gray bars) and f‐TSPCs (light gray bars) grown in 21% (solid fill) or 5% oxygen (hatched fill) (*n* = 4, mean age 9.0 years, range 1–22 years, except f‐TSPCS grown in 21% oxygen; *n* = 3, mean age 9.7 years). Error bars represent SEM. ^*^ *p* = 0.029.

By quantitative RT‐PCR there were no significant differences in the expression of stem or progenitor markers between tenocytes and f‐TDSCs (Fig. 5). Expression of thrombospondin‐4, previously reported to be a tendon‐selective marker^26^ was however significantly decreased in f‐TDSCs as compared to tenocytes grown in hypoxia.

**Figure 6:**
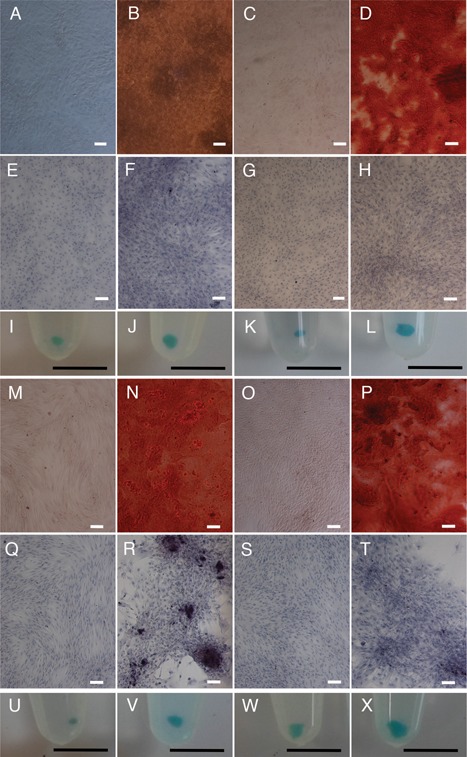
Trilineage differentiation assay for TSPCs isolated by differential adhesion onto substrates precoated with 20 μg/ml fibronectin (A–L) or by low density plating at 10 cells/cm^2^ (M–X) and grown in normoxia (A‐B, E‐F, I‐J, M‐N, Q‐R, U‐V) or 5% hypoxia (C‐D, G‐H, K‐L, O‐P, S‐T, W‐X). A–D and M–P: Osteogenic differentiation assays. TSPCs were grown in control (A, C, M, O) or osteogenic induction media (B, D, N, P) and stained with Alizarin Red. Bar; 100 μm. E–H and Q–T: Adipogenic differentiation assays. TSPCs were grown in control (E, G, Q, S) or adipogenic induction media (F, H, R, T) and stained with Oil Red O. No staining was detected. Bar; 100 μm. I–L and U–X: Chondrogenic differentiation assays. TSPC pellet cultures were grown in control (I, K, U, W) or chondrogenic induction media (J, L, V, X, and stained with Alcian Blue. Bar; 5 mm. Sample age 1 year.

Trilineage differentiation assays indicated that f‐TDSCs grown in normoxia or hypoxia were osteogenic and chondrogenic as assessed by Alizarin Red and Alcian Blue staining of pellet cultures, respectively (Fig. 6A–L). No adipogenic differentiation was detected by Oil Red O staining. Similarly, growth in hypoxic conditions did not affect the differentiation capacity of TDSCs isolated by low density plating which again differentiated to osteogenic and chondrogenic lineages, in both normoxic and hypoxic conditions, as assessed by Alizarin Red and Alcian Blue staining (Fig. 6M–X).

**Figure 7:**
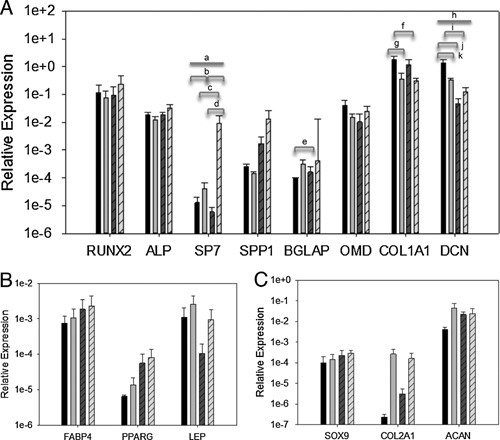
Relative expression of osteogenic (A), adipogenic (B), and chondrogenic (C) genes as compared to GAPDH for TSPCs isolated by differential adhesion onto substrates precoated with 20 μg/ml fibronectin (solid fill) or by low density plating at 10 cells/cm^2^ (hatched fill) and grown in and grown in control (dark gray bars) or osteogenic induction media (light gray bars) and 5% hypoxia. Error bars represent SEM (*n* = 3, mean age 10 years, range 1–22 years). ^a^ *p* = 0.034 (interaction), ^b^ *p* = 0.004, ^c^ *p* = 0.013, ^d^ *p* = 0.002, ^e^ *p* = 0.047, ^f^ *p* = 0.022, ^g^ *p* = 0.038, ^h^ *p* = 0.033 (interaction), ^i^ *p* = 0.002, ^j^ *p* = 0.001, ^k^ *p* = 0.022 by two‐way ANOVA.

The expression of osteogenic, adipogenic, and chondrogenic marker genes was determined for TSPCs isolated by differential adhesion to fibronectin (20 μg/ml) or by low density plating at 10 cells/cm^2^ and grown in hypoxic conditions with osteogenic, adipogenic, or chondrogenic induction media. No consistent alterations in marker gene expression were observed (Fig. 7).

## DISCUSSION

Using published methods for isolation of TSPCs by low‐density plating^8^, ^9^ we found that TSPCs from equine SDFT did not differentiate to the adipogenic lineage as assessed by Oil Red O staining. We also found that expression of osteogenic and chondrogenic marker genes was not entirely consistent with histological staining techniques for assessing differentiation. Fibronectin has been identified as a suitable substrate to maintain long term self renewal of embryonic stem cells and has been used for isolation of epidermal stem cells and cartilage progenitors.^24^, ^25^, ^27^ Fibronectin was therefore a good candidate ECM substrate for differential isolation of tendon stem cells. However, isolation of putative TSPCs by plating onto substrates pre‐coated with fibronectin, as well as growth in hypoxic conditions produced similar results to the low density plating method.

The observed restricted differentiation potential of isolated TSPCs was surprising as the equine SDFT potentially contains at least three stem cell niches; within the collagenous tendon fascicles, within the loose connective tissue of the endotenon (the interfascicular matrix) and within the vasculature present within the endotendon. Two previous studies reported adipogenic differentiation of cells from the SDFT, when comparing equine tendon cells with bone marrow stromal cells^28^ and comparing cell populations from the tendon core and peritenon.^15^ In this study, we only isolated cells from the tendon core, however, it is possible that the process of tissue transportation from the abattoir from which samples were obtained could adversely affect stem cell viability, leaving a population of progenitor cells with restricted differentiation potential. We used the same batch of serum for all experiments and it is possible that the particular batch did not support adipogenic differentiation. Alternatively the process of isolating TSPCs by low density plating or differential adhesion to fibronectin may remove a sub‐group of cells capable of adipogenic differentiation from the equine SDFT cell population. Age has been reported to affect adipogenic differentiation of rat but not human TSPCs.^29^, ^30^ In this report, we studied samples ranging from 1 to 22 years (equivalent to age 4–77 in humans) but found no observable differences in adipogenic differentiation.

We did not find significant differences between the proliferation rates of tenocytes and putative f‐TSPCs grown in 5% oxygen. However, TSPCs isolated by low‐density plating proliferated more slowly than tenocytes or f‐TSPCs in hypoxic conditions (Fig. 3) and had reduced colony forming ability (Fig. 1D). A previous report indicated that rabbit TSPCs proliferate faster than tenocytes,^10^ however, the tenocyte proliferation rates were much slower than reported here. This may be due to the presence of TSPCs in our tenocyte population, as tenocytes were isolated by direct high density plating of cells isolated from tendon. Conversely Zhang et. al. isolated tenocytes from TSPCs by subcloning all colonies from the culture dish and leaving tenocytes behind. The exercise history of the horses used in our study was unknown but exercise as well as species specific difference may also be responsible for differences in the tenocyte proliferation rates.^14^

Tendons heal slowly after injury by production of fibrotic scar tissue. Application of exogenous mesenchymal stem cells has been used to attempt to promote tendon healing, with heterogenous treatment parameters and limited outcome measures.^31^, ^32^ However, the relatively high proportion of TSPCs reported in tendon implies that tendons should have an endogenous regenerative capacity. It may be the case that TSPCs inappropriately differentiate to alternative lineages during tendon injury and aging resulting in ectopic ossification, mucoid degeneration, and fat deposition. Promoting the retention of a progenitor population with restricted differentiation potential may therefore be beneficial for tendon regeneration.

## AUTHORS' CONTRIBUTIONS

KAW, KJL, and WJEH acquired, analyzed, and interpreted data. KAW, EJVC, PDC, and EGC‐L designed the study. EGC‐L, KAW, and KJL drafted the paper. All authors critically revised the manuscript and read and approved the final submitted version.

## References

[1] O'Neil BA , Forsythe ME , Stanish WD . 2001 Chronic occupational repetitive strain injury. Can. Fam. Physician 47:311–316. 11228032 PMC2016244

[2] Hess GW . 2010 Achilles tendon rupture: a review of etiology, population, anatomy, risk factors, and injury prevention. Foot Ankle Spec. 3:29–32. 20400437 10.1177/1938640009355191

[3] Thorpe CT , Clegg PD , Birch HL . 2010 A review of tendon injury: why is the equine superficial digital flexor tendon most at risk?. Equine Vet. J. 42:174–180. 20156256 10.2746/042516409X480395

[4] Kasashima Y , Takahashi T , Smith RKW . et al. 2004 Prevalence of superficial digital flexor tendonitis and suspensory desmitis in Japanese Thoroughbred flat racehorses in 1999. Equine Vet. J. 36:346–350. 15163043 10.2746/0425164044890580

[5] Thorpe CT , Riley GP , Birch HL . et al. 2014 Fascicles from energy‐storing tendons show an age‐specific response to cyclic fatigue loading. J. R. Soc. Interface 11:20131058. 24402919 10.1098/rsif.2013.1058 PMC3899876

[6] Thorpe CT , Udeze CP , Birch HL . et al. 2013 Capacity for sliding between tendon fascicles decreases with ageing in injury prone equine tendons: a possible mechanism for age‐related tendinopathy?. Eur. Cell. Mater. 25:48–60. 23300032 10.22203/ecm.v025a04

[7] Peffers MJ , Thorpe CT , Collins JA . et al. 2014 Proteomic analysis reveals age‐related changes in tendon matrix composition, with age‐ and injury‐specific matrix fragmentation. J. Biol. Chem. 289:25867–25878. 25077967 10.1074/jbc.M114.566554 PMC4162187

[8] Bi Y , Ehirchiou D , Kilts TM . et al. 2007 Identification of tendon stem/progenitor cells and the role of the extracellular matrix in their niche. Nat. Med. 13:1219–1227. 17828274 10.1038/nm1630

[9] Rui YF , Lui PP , Li G . et al. 2010 Isolation and characterization of multipotent rat tendon‐derived stem cells. Tissue Eng. Part A 16:1549–1558. 20001227 10.1089/ten.TEA.2009.0529

[10] Zhang J , Wang JH . 2010 Characterization of differential properties of rabbit tendon stem cells and tenocytes. BMC Musculoskelet. Disord. 11:10. 20082706 10.1186/1471-2474-11-10 PMC2822826

[11] Zhang J , Wang JH . 2010 Platelet‐rich plasma releasate promotes differentiation of tendon stem cells into active tenocytes. Am. J. Sports Med. 38:2477–2486. 20802092 10.1177/0363546510376750

[12] Mienaltowski MJ , Adams SM , Birk DE . 2013 Regional differences in stem cell/progenitor cell populations from the mouse achilles tendon. Tissue Eng. Part A. 19:199–210. 22871316 10.1089/ten.tea.2012.0182 PMC3530943

[13] Mendias CL , Gumucio JP , Bakhurin KI . et al. 2012 Physiological loading of tendons induces scleraxis expression in epitenon fibroblasts. J. Orthop. Res. 30:606–612. 21913219 10.1002/jor.21550 PMC3245815

[14] Zhang J , Pan T , Liu Y . et al. 2010 Mouse treadmill running enhances tendons by expanding the pool of tendon stem cells (TSCs) and TSC‐related cellular production of collagen. J. Orthop. Res. 28:1178–1183. 20225313 10.1002/jor.21123

[15] Cadby JA , Buehler E , Godbout C . et al. 2014 Differences between the cell populations from the peritenon and the tendon core with regard to their potential implication in tendon repair. PLoS One 9:e92474. 24651449 10.1371/journal.pone.0092474 PMC3961373

[16] Benjamin M , Kaiser E , Milz S . 2008 Structure‐function relationships in tendons: a review. J. Anat. 212:211–228. 18304204 10.1111/j.1469-7580.2008.00864.x PMC2408985

[17] Lee WY , Lui PP , Rui YF . 2012 Hypoxia‐mediated efficient expansion of human tendon‐derived stem cells in vitro. Tissue Eng. Part A 18:484–498. 21943340 10.1089/ten.tea.2011.0130 PMC3286812

[18] Zhang Y , Wang B , Zhang WJ . et al. 2010 Enhanced proliferation capacity of porcine tenocytes in low O2 tension culture. Biotechnol. Lett. 32:181–187. 19821074 10.1007/s10529-009-0137-8

[19] Zhang J , Wang JH . 2013 Human tendon stem cells better maintain their stemness in hypoxic culture conditions. PLoS One 8:e61424. 23613849 10.1371/journal.pone.0061424 PMC3629026

[20] PromoCell:Application‐Notes. Date accessed: November 2014. Osteogenic Differentiation and Analysis of MSC. http://www.promocell.com/fileadmin/knowledgebase/pdf-xls/Osteogenic_Differentiation_and_Analysis_of_MSC.pdf.

[21] PromoCell:Application‐Notes. Date accessed: November 2014. Adipogenic Differentiation and Analysis of MSC. http://www.promocell.com/fileadmin/knowledgebase/pdf-xls/Adipogenic_Differentiation_and_Analysis_of_MSC.pdf.

[22] PromoCell:Application‐Notes. Date accessed: November 2014. Chondrogenic Differentiation and Analysis of MSC. http://www.promocell.com/fileadmin/knowledgebase/pdf-xls/Chondrogenic_Differentiation_and_Analysis_of_MSC.pdf.

[23] Schmittgen TD , Livak KJ . 2008 Analyzing real‐time PCR data by the comparative CT method. Nat. Protoc. 3:1101–1108. 18546601 10.1038/nprot.2008.73

[24] Dowthwaite GP , Bishop JC , Redman SN . et al. 2004 The surface of articular cartilage contains a progenitor cell population. J. Cell Sci. 117:889–897. 14762107 10.1242/jcs.00912

[25] Jones PH , Watt FM . 1993 Separation of human epidermal stem cells from transit amplifying cells on the basis of differences in integrin function and expression. Cell 73:713–724. 8500165 10.1016/0092-8674(93)90251-k

[26] Jelinsky SA , Archambault J , Li L . et al. 2010 Tendon‐selective genes identified from rat and human musculoskeletal tissues. J. Orthop. Res. 28:289–297. 19780194 10.1002/jor.20999

[27] Baxter MA , Camarasa MV , Bates N . et al. 2009 Analysis of the distinct functions of growth factors and tissue culture substrates necessary for the long‐term self‐renewal of human embryonic stem cell lines. Stem Cell Res. 3:28–38. 19428319 10.1016/j.scr.2009.03.002

[28] Lovati AB , Corradetti B , Lange Consiglio A . et al. 2011 Characterization and differentiation of equine tendon‐derived progenitor cells. J. Biol. Regul. Homeost. Agents 25:S75–S84. 22051173

[29] Zhou Z , Akinbiyi T , Xu L . et al. 2010 Tendon‐derived stem/progenitor cell aging: defective self‐renewal and altered fate. Aging Cell 9:911–915. 20569237 10.1111/j.1474-9726.2010.00598.x PMC2944918

[30] Kohler J , Popov C , Klotz B . et al. 2013 Uncovering the cellular and molecular changes in tendon stem/progenitor cells attributed to tendon aging and degeneration. Aging Cell 12:988–999. 23826660 10.1111/acel.12124 PMC4225469

[31] Lui PP , Ng SW . 2013 Cell therapy for the treatment of tendinopathy‐a systematic review on the pre‐clinical and clinical evidence. Semin. Arthritis Rheum. 42:651–666. 23369658 10.1016/j.semarthrit.2012.10.004

[32] Reed SA , Leahy ER . 2013 Growth and Development Symposium: Stem cell therapy in equine tendon injury. J. Anim. Sci. 91:59–65. 23100589 10.2527/jas.2012-5736

